# With a Little Help From My Family: A Mixed-Method Study on the Outcomes of Family Support and Workload

**DOI:** 10.5964/ejop.v12i4.1159

**Published:** 2016-11-18

**Authors:** Alessandro Lo Presti, Fulvia D’Aloisio, Sara Pluviano

**Affiliations:** a Department of Psychology, Second University of Naples, Caserta, Italy; b Suor Orsola Benincasa University, Napoli, Italy; c University of Edinburgh, Edinburgh, United Kingdom; The Maria Grzegorzewska University, Warsaw, Poland

**Keywords:** family-to-work enrichment, mixed-method approach, family support, family workload, gender issues, work-family enrichment

## Abstract

Our aim was to investigate some predictors and outcomes of family-to-work enrichment (FWE) via a mixed-method approach. We sampled 447 married employees of an Italian factory. Survey results from Study 1 showed that emotional support from family positively predicted FWE, while this latter mediated the associations between the former on one side, and work engagement and life satisfaction on the other. Moreover, extra-household support directly associated positively with life satisfaction. Evidence from 20 anthropological in-depth interviews (Study 2) returned a more complex picture, highlighting the gendered role of partners inside couples, the importance of kinship support, the sense and the value of filiation and parenthood in their connection with job roles, the complex and continuous interplay between family and life domains. In combination, results from both studies stressed the importance of family support; additionally, evidences from Study 2 suggested that FWE could be better understood taking into account crossover dynamics and the compresence of work-to-family enrichment and conflict. In sum, these studies contributed to shed light on FWE dynamics, an under-researched topic in Italy, whose knowledge could be of great empirical and practical value.

The current global trend of increased dual-earner and single-parent families, coupled with a blurring of gender roles, provides a vivid illustration of how work and non-work aspects of life are intertwined (Greenhaus & Powell, 2006). Greenhaus and Powell (2006) provided a theory that specifies the conditions under which “work and family roles are ‘allies’ rather than ‘enemies’” (p. 72), thus beginning to test the idea of *work-family enrichment*, defined as the extent to which experiences in Role A (work or family) improve the quality of life, namely heighten high performance or positive affect, in Role B (family or work). Enrichment can occur bi-directionally, meaning that work can provide resource gains that have a positive impact on the family role (*work-to-family enrichment*, WFE) or, conversely, family can provide resource gains that lead to positive impact on the work role (*family-to-work enrichment*, FWE). For clarity, in this paper we specifically emphasised this latter direction (i.e., from family to work) that, in spite of its significance, appeared for a long time as “the neglected side of the work-family interface” (Crouter, 1984, p. 425). According to a meta-analysis by McNall, Nicklin, and Masuda (2010) carried out on 25 previous studies, FWE was associated with job satisfaction, affective commitment, family satisfaction, physical and mental health. Other studies found significant associations with life satisfaction (Lazarevic, Holman, Oswald, & Kramer, 2015), organizational citizenship behaviours (Bhargava & Baral, 2009), positive mood and psychological distress (Carlson, Hunter, Ferguson, & Whitten, 2014), among others. It derives that FWE does not only represent an important variable in order to explain the positive interplay between family and work domains but is accompanied by a large variety of significant work and general positive outcomes of practical and organizational salience. Nevertheless, the amount of empirical evidence about FWE (in particular, about its predictors), as compared to WFE and other work-family variables, remains limited (Mauno & Rantanen, 2013), and almost absent as regards to Italy. Consistently with the importance of co-workers support within organizational contexts, especially for work-to-family enrichment (Lo Presti & Mauno, 2016), family support, and workload, seem to be fundamental variables that can foster, or hinder, the development of family-to-work enrichment, to the extent that they can provide individuals with valuable psychosocial resources that can facilitate their subsequent work experiences. Despite the importance of this topic, scholars have usually used comprehensive measures of family support (Bhargava & Baral, 2009; Lu, 2011), returning a simplified picture of this variable, and neglected to evaluate family workload. Finally, given the complexity of this issue, which connects to the intertwinement between family and work domains, a quali-quantitative approach would be preferable; yet it seems that scholars have preferred up to now only quantitative approaches, overlooking more comprehensive research designs.

Stemming from this rationale, this work challenges and expands the extant research on FWE through a mixed-method approach. First, consistently with work-family enrichment theoretical frameworks (Greenhaus & Powell, 2006; Wayne, Grzywacz, Carlson, & Kacmar, 2007), as well as previous empirical evidence, we quantitatively evaluated the impact of different facets of family support, and family workload, as antecedents of FWE, and their combined effects on life satisfaction and work engagement (Study 1) on a sample of blue-collar workers, an under-represented occupational group in work-family studies. Second, based on current evidence concerning the well-known Italian gap between male and female labour rates, and the impact of its specific welfare system (the so-called *familistic welfare*) on work-family conciliation, we qualitatively examined, through an anthropological in-depth perspective, the interplay between work and family life and their resulting effects among blue-collar workers, contrasting them with Study 1 evidence (Study 2).

From a practical point of view, knowing which family variables, in terms of family workload and facets of family support, have a positive impact on FWE, and thus on work engagement and life satisfaction, could provide practitioners and HR professionals with useful information about what to target with interventions aimed at improving individuals’ work-family interface and consequently their quality of life, both at work and in general. Nevertheless, it must be acknowledged that higher life satisfaction and work engagement can have a positive impact on individual motivation and, thus, lead to heightened work performance; it derives that promoting family-to-work enrichment does not only go in the interest of employees but of the organization itself.

## Theoretical Foundation

Crucial driver of the enrichment process is the generation of resources, conceptualized as assets that individuals may capitalize to solve a problem or manage challenging situations, therefore keeping their life manageable (Greenhaus & Powell, 2006). These include skills and perspectives, psychological and physical resources, social-capital resources, flexibility, and material resources.

Wayne et al. (2007) proposed the *Resource-Gain-Development perspective* of work-family enrichment based on the Conservation of Resources theory (Hobfoll, 2011). They defined resources as characteristics of the environment that (Bakker & Demerouti, 2007): (a) support individuals in achieving goals, (b) address demands (i.e., aspects of the job that require sustained effort, such as high work pressure), and/or (c) encourage personal growth and development. Wayne et al. (2007) argued that “the basic premise of the Resource-Gain-Development perspective is that individuals have natural tendencies to grow, develop, and achieve the highest levels of functioning for themselves and the systems in which they participate including families and organizations” (p. 66). According to this model, enrichment acts as a mediator between resources and work/family outcomes. Thus, resources may increase enrichment between work and family domains, which in turn may have positive outcomes, both at work and home (Hunter, Perry, Carlson, & Smith, 2010). Consistently with this perspective, social resources (e.g., social support from family members) promote family-to-work enrichment by fostering a positive family environment that equips employees with sufficient resources to successfully and satisfactory functioning in the work domain.

Furthermore, consistently with McNall et al. (2010), it is also possible to expect that enrichment would be associated with outcomes related to the domain from which it firstly originated. Thus, for instance, it is plausible to assume that FWE could also be associated with life and family satisfaction, apart from job satisfaction and work engagement (as it would be assumable from its original theoretical foundation). As Wayne, Musisca, and Fleeson (2004) explained, “it may be that when individuals make attributions about the benefits of one role to the other, this primarily results in more positive affect and behavioral investment in the role seen as providing the benefit” (p. 124).

## Family Demands and Resources

As we previously stated, we focused on family workload (as a family demand) and different facets of family support based on the fact that a comprehensive examination of their effects on FWE is still lacking in the literature on work-family enrichment, and that examining such phenomena could have direct positive practical implications for organizations, such as tailoring work-family interventions targeting also the home domain in order to enhance family support dynamics.

In the arenas of work and family domains, a large body of research focuses on the negative home-work interaction (Long Dilworth & Kingsbury, 2005), referred as the detrimental load effects that have built up in the home situation and interfere with functioning at work. Indeed, higher home demands, measured as general household activities (Demerouti, Geurts, & Kompier, 2004) or in terms of number and age of children (Voydanoff, 1988), are usually associated with a higher level of negative home-work interaction.

However, according to Greenhaus and Powell (2006), the tug of war between work and family may be loosened by the acquisition of *social capital*, defined as “the goodwill that is engendered by the fabric of social relations and that can be mobilized to facilitate action” (Adler & Kwon, 2002, p. 17). Thus, social support received from a family member must be regarded as a key resource that, besides leading someone to feel “cared for and loved, esteemed and valued, and a member of a network of mutual obligation” (Cobb, 1976, p. 300), may also foster affect in the family that can be transferred to the individual’s functioning at work.

Traditionally, two types of non-work social support have received empirical validation: (a) *emotional* or *socioemotional*, and (b) *instrumental* or *tangible*. The former involves the family’s interest in the employee’s work and those general behaviours or attitudes of care and concern that spread in one’s mind encouragement, attention and positive regard, understanding, and guidance with problem-solving. On the other hand, the latter encompasses those family member behaviours and attitudes specifically aimed at facilitating the employee’s household tasks and responsibilities, as well as at organizing family life as to accommodate the employee’s work schedule or job requirements (King, Mattimore, King, & Adams, 1995).

It might also be noted that a great deal of attention in the literature has been paid to spouse support (Carlson & Perrewé, 1999) but it should also be underlined the important role of other forms of non-work social support on whom a person can rely on. For instance, in wider families individuals may lean on other domestic support resources such as elderly parents or domestic helpers, who may help to mitigate the stress within the family domain (Pagani & Marenzi, 2008). Moreover, the employee may also benefit of the helping hand of people who are not bounded by permanence or sharing of a household, such as other kin or friends. To comprehensively account for the variety of non-work social support but also avoid any overlap between its different forms, in this study we specifically investigated the roles of family (spouse, sons, and daughters) and extra-household social support.

Several findings corroborate the relationship between family support and FWE. Aryee, Srinivas, and Tan (2005) used a comprehensive measure of family support, encompassing instrumental and emotional sources of support. Their findings speak in favour of a positive relationship between family support and FWE, suggesting that supportive family experiences may allow individuals to work longer hours and avail them of developmental opportunities.

In contrast, Wayne, Randel, and Stevens (2006) found that only family emotional support strongly predicted FWE. This suggests that, in order to generate positive experiences across domains, feeling that family members care about individual’s work concerns, rather than sharing household responsibilities, is of a greater importance.

Especially noteworthy is a longitudinal study carried out by Hakanen, Peeters, and Perhoniemi (2011) that, instead of investigating only one form of support, proposed a wider conceptualization of home resources, conceived as both the emotional, instrumental and appraisal support from one’s family/partner, as well as the social support from one’s friend. In particular, the authors pointed out that FWE positively predicted both home resources and marital satisfaction over time.

Nevertheless, we believe that this evidence unveil just the tip of the iceberg of how studying the importance of family support might enhance our view of enrichment dynamics. Therefore, on the call to incorporate both demand- and resource-family factors in examining FWE, we hypothesized that:

*H1*: Family workload will be negatively related to FWE.

*H2a-c*: Family support, including both emotional (a) and instrumental (b) family social support, as well as extra-household support (c), will be positively related to FWE.

## Mediation Hypotheses Concerning FWE

This study attempted to examine some specific work-related (work engagement) and non-work (life satisfaction) outcomes of positive home-work interaction. In this vein, consistently with the Resource-Gain-Development perspective (Wayne et al., 2007), that assumes that resources may increase enrichment between work and family domains, which then may improve satisfaction at work and at home, FWE could represent a key mediator between family resources and demands on one side, and home/work outcomes on the other.

Siu et al. (2010) clarified how work engagement might represent a crucial factor in testing Greenhaus and Powell’s (2006) dual pathways model. In this respect, family support should be considered as a key resource that could enhance work engagement. Indeed, employees may leverage instrumental advice and affective resources received from family to reach their craved work goals, or family support may also motivate a member to work harder by providing love and care (Grzywacz & Marks, 2000). In addition, Rothbard (2001), examining whether engagement in one role was enriching or depleting to the engagement in the other role, found that (though only among women) positive family affect related to a greater work absorption. Finally, Hakanen et al. (2011) revealed that work-to-family enrichment related to work engagement and these variables reciprocally influenced each other over time. The opposite might also be true, namely FWE can predict work engagement. Indeed, albeit it is largely demonstrated that both directions of enrichment may be related to work outcomes, it could be also the case that enrichment operates similarly to conflict, implying that work-to-family enrichment is more closely tied with family outcomes, whereas FWE is more closely related to work outcomes (McNall et al., 2010).

Based on the above arguments, we propose that:

*H3a-c*: Family support, including both emotional (a) and instrumental (b) family social support, as well as extra-household support (c), will be positively related to work engagement via FWE.

As several studies linked work-to-family conflict to lower life satisfaction (e.g., Allen, Herst, Bruck, & Sutton, 2000; Eby, Casper, Lockwood, Bordeaux, & Brinley, 2005), it is not so surprising to expect the reverse; that is, evidence suggesting the relationship between enrichment and life satisfaction (e.g., McNall et al., 2010), although not so convincingly as it is for work-to-family conflict (Mauno, Kinnunen, & Rantanen, 2011). For instance, van Steenbergen, Ellemers, and Mooijaart (2007) revealed that, if the experience of conflict was highly predictive of stress-related outcomes (e.g., emotional exhaustion), the inclusion of a similar construct to enrichment, namely *facilitation* (i.e., individual’s judgement that participation in one role makes participation in another role easier, p. 281), significantly and substantially improved the prediction of positive work outcomes (e.g., affective commitment) as well as non-work outcomes (e.g., life satisfaction), over and above the effects of conflict. Also Mauno et al. (2011) underlined that FWE was a strong predictor of life satisfaction.

In addition, according to Greenhaus and Powell (2006), family participation is likely to generate a variety of resources (e.g., social-capital resources), which can bolster one’s performance and positive affect at home, and in turn improve one’s positive affect at work. Thus, it follows that greater positive feelings about own family role might subsequently result in reciprocation in the form of higher family and life satisfaction in general (McNall et al., 2010). Indeed, involvement with family domain has been linked to a greater life satisfaction (Judge, Boudreau, & Bretz, 1994). For instance, Adams, King, and King (1996) revealed that high levels of family involvement were associated with higher levels of emotional support from family members, which, in turn, had a positive relationship with life satisfaction. Similarly, Greenhaus, Collins, and Shaw (2003) showed how quality of life was highest for those more engaged or more satisfied in family than work, and lowest for those who are more engaged or more satisfied in work than family. Also, Bhargava and Baral (2009) provided evidence for the positive relationship between family support and FWE and also found that FWE related to family and job satisfaction. Finally, as previously mentioned, consistently with McNall et al. (2010), it is also possible to expect that enrichment would be associated with outcomes related to other domains than work.

Based on the above arguments, we propose that:

*H4a-c*: Family support, including both emotional (a) and instrumental (b) family social support, as well as extra-household (c), will be positively related to life satisfaction via FWE.

**Figure 1:**
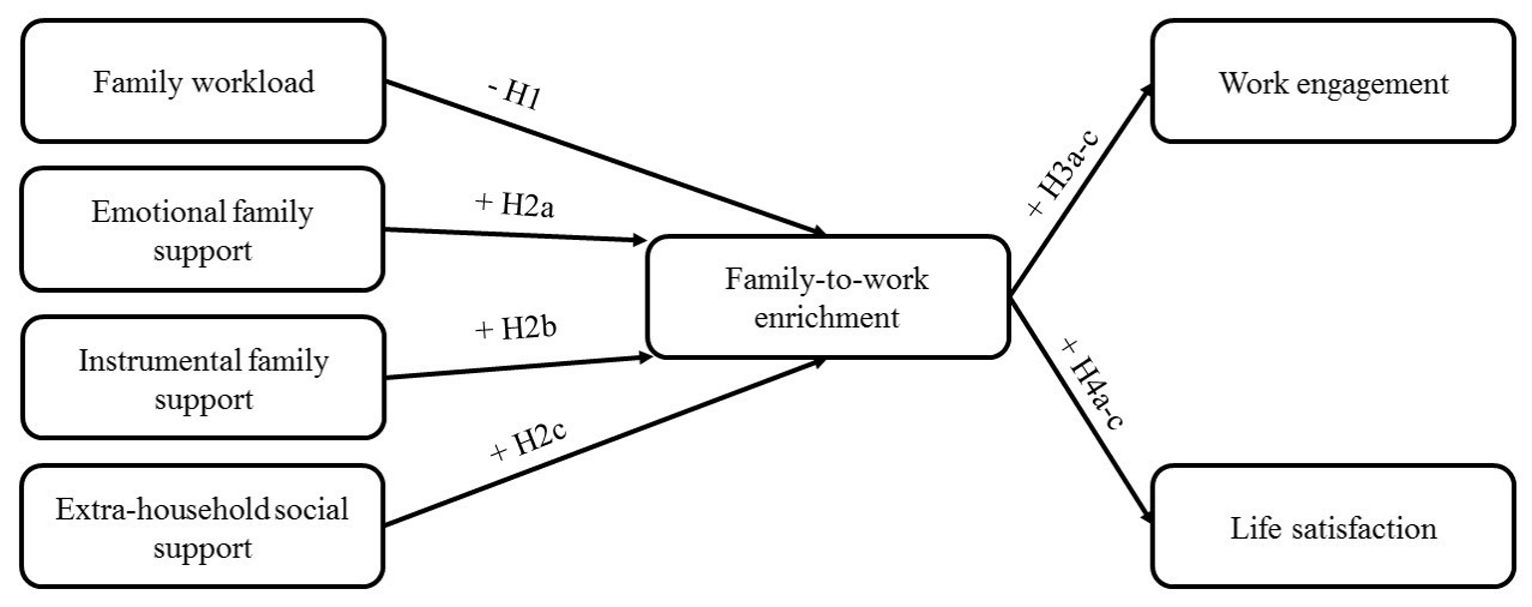
Research model.

Figure 1 depicts our research model.

## Study 1

### Method

#### Participants

Data were collected by means of a self-report questionnaire among employees of a food-processing factory located in Southern Italy, one of the main productive sector in that part of the country.

Each questionnaire was distributed inside a sealable envelope (accompanied by a letter of invitation to participate in the study) and retrieved by a survey researcher. An estimated plant population was about 1000 employees (fluctuating according to seasonal production needs), and we randomly sampled the 60% of the total plant population, thus 600 employees. A random sampling was carried out in order to have a representative sample of the total plant population and not interfere too much with organizational processes. This sampling procedure was negotiated with managers. 530 questionnaires were returned (88.3% response rate), and, of these, 489 were valid for research purposes (the other 41 were mostly incomplete). After applying a *post-hoc* selection criterion, selecting married or cohabitating employees, 447 employees were retained for the subsequent analyses. We decided to focus on partnered respondents owing to their particular relevance for work-family research, given the scarcity of other family status (only 10%), and in order to have a more homogeneous sample.

The sample comprised 370 (82.8%) men and 77 women (17.2%), and was consistent with the overall situation in Italy regarding gender distribution in industrial manufacturing organizations (ISTAT, 2013). Mean sample age was 41.64 years (*SD* = 7.01; *Min.* = 25, *Max.* = 60), and the average number of children per household was 1.64 (*SD* = .94; *Min.* = 0, *Max.* = 5). Most participants (*n* = 385, 86.1%) had a permanent contract and were shift workers (*n* = 388, 86.8%). The great majority of the participants (*n* = 395, 88.4%) worked in blue-collar occupations.

#### Measures

The measures were initially translated from English to Italian, and have already been used in previous Italian studies (Lo Presti & Nonnis, 2011), where they have turned out to be reliable measures of the studied phenomena.

*Family workload* was assessed through an 8-item scale developed for the present study (Cronbach’s α = .85; *M* = 16.38, *SD* = 7.43) that asked, with reference to a typical week, to evaluate the frequency according to which the individual was in charge to accomplish a series of family chores within own household (e.g., “do grocery shopping”, “prepare a hot meal”, etc.). The complete list of items is available from the first author upon a request. Responses were based on a five-point Likert scale (from 0 = never to 4 = always) while scores ranged between 0 and 32.

*Emotional and instrumental family social support* were assessed via the Family Support Inventory by King et al. (1995). Emotional (e.g., “members of my family are interested in my job”; Cronbach’s α = .80; *M* = 22.9, *SD* = 4.96) and instrumental (e.g., “my family leaves too much of the daily details of running the house to me”; Cronbach’s α = .75; *M* = 23.1, *SD* = 4.9) family support scales both featured 6 items. Responses were based on a five-point Likert scale (from 1 = completely false to 5 = completely true) while scores ranged between 6 and 30.

*Extra-household social support* was evaluated through a single item (e.g., “When I need it, I am confident I can count on extra help from my relatives as parents, uncles, cousins, etc. to accomplish my family chores”; *M* = 3.41, *SD* = 1.4). Responses were based on a five-point Likert scale (from 1 = completely false to 5 = completely true).

*Family-to-work enrichment,* was assessed by using 6 items (e.g., “My involvement in my family requires me to avoid wasting time at work and this helps me be a better worker”) from the Work Family Enrichment Scale by Carlson, Kacmar, Wayne, and Grzywacz (2006; Italian adaptation by Ghislieri, Martini, Gatti, & Colombo, 2011; Cronbach’s α = .92; *M* = 21, *SD* = 6.18). Responses were given on a five-point Likert scale (from 1 = completely disagree to 5 = completely agree) while scores ranged between 6 and 30.

*Work engagement* was evaluated using the Utrecht Work Engagement Scale (Schaufeli, Bakker, & Salanova, 2006). It comprised 9 items (e.g., “At work, I feel bursting with energy”; Cronbach’s α = .94; *M* = 31.77, *SD* = 14.07); responses were based on a seven-point Likert scale (from 0 = never to 6 = always) while scores ranged between 0 and 54.

*Life satisfaction* was assessed via the Satisfaction with Life Scale by Diener, Emmons, Larsen, and Griffin (1985). The scale comprised 5 items (e.g., “I am satisfied with my life”; Cronbach’s α = .88; *M* = 15.52, *SD* = 5.25). Responses were based on a five-point Likert scale (from 1 = completely false to 5 = completely true) while scores ranged between 5 and 25.

Some background factors were used as control variables in this study: sex (1 = male, 2 = female), age, number of children, and family/job involvement (one item: ranging from -5 = my job is the most important thing to me to 5 = my family is the most important thing to me). These variables were controlled for in the analyses because they have found to be relevant in work-family interface research (Byron, 2005; Mauno, Kinnunen, & Ruokolainen, 2006).

#### Data Analysis

Descriptive statistics (mean and standard deviations) and zero-order correlations were used to describe variables and their inter-correlations. Hierarchical linear regression analysis was used to test the associations between predictors and the three outcomes (FWE, work engagement, life satisfaction). Mediation effects were tested through bootstrapping (Hayes, 2013).

### Results

Table 1 depicts zero-order correlations between study variables.

**Table 1:** Descriptive Statistics and Zero-Order Correlations (Under the Diagonal) Between the Study Variables and Their Standard Errors (Above the Diagonal)

Variables	*M* (*SD*)	1	2	3	4	5	6	7	8	9	10	11
1. Gender ^a^	-	-	.05	.05	.05	.03	.05	.04	.05	.05	.05	.05
2. Age	41.63 (7)	-.17	-	.04	.05	.05	.05	.05	.05	.05	.05	.05
3. Number of sons	1.64 (.94)	-.13	.28	-	.05	.05	.05	.05	.05	.05	.05	.05
4. Job/Family involvement ^b^	3.14 (1.99)	.15	-.08	.07	-	.05	.05	.05	.05	.05	.05	.05
5. Family workload	16.38 (7.43)	.67	-.22	-.15	.15	(.85)	.05	.04	.05	.05	.05	.05
6. Emotional family support	22.9 (4.96)	-.12	-.06	0	-.01	-.14	(.80)	.04	.04	.04	.04	.05
7. Instrumental family support	23.1 (4.9)	-.34	.05	.07	-.02	-.35	.59	(.75)	.04	.05	.05	.05
8. Extra-household support	3.41 (1.4)	-.04	-.07	-.07	.08	-.02	.38	.35	-	.05	.05	.04
9. Family-to-work enrichment	21 (6.18)	.02	-.01	-.06	-.06	-.02	.37	.16	.17	(.92)	.04	.04
10. Work engagement	31.77 (14.07)	-.07	.13	.01	-.20	-.04	.26	.14	.11	.41	(.94)	.04
11. Life satisfaction	15.52 (5.25)	-.03	-.05	-.06	-.07	-.01	.25	.14	.34	.35	.46	(.88)

*Note.* ^a^1 = male, 2 = female. ^b^lower scores mean higher job involvement while higher scores mean higher family involvement; Cronbach alphas in brackets on the diagonal.

Family workload did not correlate with none of the three outcomes. Emotional family support positively correlated with FWE (*r* = .37, *p* < .01), work engagement (*r* = .26, *p* < .01) and life satisfaction (*r* = .25, *p* < .01). Instrumental family support positively correlated with FWE (*r* = .16, *p* < .01), work engagement (*r* = .14, *p* < .01) and life satisfaction (*r* = .14, *p* < .01). Extra-household support positively correlated with FWE (*r* = .17, *p* < .01), work engagement (*r* = .11, *p* < .05) and life satisfaction (*r* = .34, *p* < .01). Finally, FWE positively correlated with work engagement (*r* = .41, *p* < .01) and life satisfaction (*r* = .35, *p* < .01).

The results of the hierarchical linear regression analysis on FWE are shown in Table 2.

**Table 2:** Results of Hierarchical Linear Regression Analysis for Family-To-Work Enrichment, Work Engagement and Life Satisfaction

Variables	Family-to-work enrichment			Work engagement				Life satisfaction			
	β_Step 1_	β_Step 2_	β_Step 3_	β_Step 1_	β_Step 2_	β_Step 3_	β_Step 4_	β_Step 1_	β_Step 2_	β_Step 3_	β_Step 4_
Gender ^a^	.02	.10	.10	.01	.03	.03	.01	-.04	-.03	-.04	-.07
Age	.01	-.01	.03	.14*	.14*	.16**	.15**	-.07	-.07	-.04	-.05
Children	-.05	-.05	-.05	-.03	-.03	-.02	-.01	-.04	-.04	-.02	-.01
Salience ^b^	-.07	-.07	-.07	-.19***	-.19***	-.19***	-.17***	-.08	-.08	-.10*	-.08
Family-workload		-.12	-.06		-.03	.01	.03		-.01	.01	.03
Emotional family support			.37***			.21**	.11			.15*	.06
Instrumental family support			-.05			-.02	-.01			-.12	-.10
Extra-household support			.03			.06	.05			.31***	.31***
Family-to-work enrichment							.28***				.25***
*R*^2^	.01	.01	.14***	.06***	.06	.11***	.17***	.01	.01	.14***	.19***
∆*R*^2^			.12***			.05***	.07***			.12***	.06***

*Note.* ^a^1 = male, 2 = female. ^b^lower scores mean higher job involvement while higher scores mean higher family involvement.

*p < .05. **p < .01. ***p < .001.

Control variables (i.e., gender, age, number of children, job/family salience) were inserted at Step 1, family workload (as a home demand) at Step 2, while emotional, instrumental and extra-household support (as home resources) were inserted at Step 3.

Neither control variables nor family workload did show any significant association with FWE. Emotional family support did show a significant positive association (β = .37, *p* < .001). *R*^2^ was equal to 14% (*p* < .001), the majority of it (12%, *p* < .001) attributable to home resources (i.e., emotional family support).

Table 2 also shows the results of hierarchical linear regression analyses on work engagement and life satisfaction, respectively. Steps 1 through 3 were identical to the regression on FWE, while the two following regressions included an additional fourth step, featuring the insertion of FWE as ultimate predictor.

As regards to work engagement, age showed similar β coefficients across the four steps (ranging between β_Step 1_ = .14, *p* < .05 and β_Step 3_ = .16, *p* < .01). The same happened for job/family salience (ranging between β_Step 4_ = -.17, *p* < .001 and β_Step 1-3_ = -.19, *p* < .001), meaning that those more involved in their job (instead of family) show higher work engagement. Emotional family support showed a positive association (β = .21, *p* < .01) at Step 3, while its coefficient lost statistical significance after the insertion of FWE (β = .28, *p* < .001) at Step 4. Control variables explained the 6% (*p* < .001) of work engagement variance, 5% of variance (*p* < .001) was attributable to home resources and 7% (*p* < .001) to FWE. Total *R*^2^ was equal to 17% (*p* < .001).

As regards to life satisfaction, emotional family support showed a significant positive association (β = .15, *p* < .05) only at Step 3, while extra-household support showed the same coefficient (β = .31, *p* < .001) across Steps 3 and 4. Finally, FWE showed a positive significant coefficient (β = .25, *p* < .001). Home resources explained the 12% (*p* < .001) of life satisfaction variance, while FWE added more 6% (*p* < .001). Total *R*^2^ was equal to 19% (*p* < .001).

Finally, the decrease in emotional family support’s beta coefficient between Steps 3 and 4 could be an indicator of a potential mediation effect by FWE towards work engagement and life satisfaction, respectively. We verified mediation through bootstrapping (1000 samples; CI = 95%) and using the PROCESS macro by Hayes (2013). As regards to work engagement, the unstandardized effect of emotional support before entering FWE, *B* = .45, *p* < .001, 95% CI [.35, .56], suffered a decrease after the insertion of this latter variable, *B* = .35, *p* < .01, 95% CI [.09, .61]. The indirect effect of emotional support through FWE was significant, *B* = .37, *p* < .01, 95% CI [.25, .53] and testified of a partial mediation. As regards to life satisfaction, the unstandardized effect of emotional support before entering FWE, *B* = .45, *p* < .001, 95% CI [.35, .56] suffered a decrease after the insertion of this latter variable, *B* = .15, *p* < .01, 95% CI [.05, .25]. The indirect effect of emotional support through FWE was significant, *B* = .12, *p* < .01; 95% CI [.07, .17] and testified of a partial mediation.

### Discussion

Based on premises of the theoretical work of Greenhaus and Powell (2006) as well as Wayne et al. (2007), this study complied with recent calls to rely on a more balanced view of the work-family interface (Voydanoff, 2004), by investigating first how family workload negatively related to FWE (H1) and, conversely, emotional (H2a) and instrumental (H2b) family social support, as well as extra-household support (H2c), positively predicted FWE, and consequently work engagement and life satisfaction.

However, counterintuitively, H1 was not supported as family workload did not negatively affect FWE. This evidence could be explained taking into account that our sample was male-dominated and that, still today, in Italy (especially the Southern part, mainly due to cultural reasons) the burden of family chores is largely upon women (see also Study 2). Additionally, among the different assumptions about the positive relationship between overall family support and FWE, only the first one concerning the positive affect of emotional family support (H2a) was confirmed. Thus, consistently with Wayne et al. (2006), it seems that in many if not most individual’s experiences of enrichment a key role is played by those family behaviors and attitudes of whole-hearted care, love, understanding, guidance, and so on, rather than just concerns about sharing household responsibilities. Interestingly, extra-household support, although did not show any significant association with FWE, directly associated positively with life satisfaction. It could be the case that having the support of a kin is an important asset for everyday family life’s arrangement, thus contributing to a greater global life satisfaction, but this does not necessarily improve one’s functioning at work. In any case, our study demonstrated that different typologies of support (i.e., emotional and instrumental), as well as diverse sources of support, such as that one received from other people outside the narrow burdens of family (e.g., other kin or friend), should not be collapsed into an aggregate measure of overall non-work support because they can differently relate to enrichment.

The mediation analyses also showed interesting results. Regarding to specific work outcomes, partially consistent with our expectations, FWE mediated the relationship between emotional support and work engagement (H3a) but did not similarly mediate the relationship between instrumental family support (H3b) nor extra-household support (H3c) on one hand and work engagement on the other hand. The same is true as regards to the other outcome; indeed, FWE mediated the relationship between emotional support and life satisfaction (H4a) but did not mediate the relationship between instrumental family support (H4b) nor extra-household support (H4c) on one hand and life satisfaction on the other. Thus, the present study points to family emotional support, via positive affect generated at home that transfers to work, a key role in driving specific positive general and work outcomes. Further research should continue to examine the influence of enrichment on other organizationally relevant outcomes (e.g., turnover intentions, job performance), as well as non-work related variables (e.g., physical and mental health).

Anyway, in the current study there are a number of limitations that must be acknowledged. Firstly, the data was based on a single source, which may inflate common method variance. Future research should consider the possibility of capturing perceptions of FWE from more than one source (e.g., spouse, co-worker) to mitigate this effect. Furthermore, the study was cross-sectional and certainly future research might benefit from longitudinal data (Casper, Eby, Bordeaux, Lockwood, & Lambert, 2007) to disentangle the direction of causality and mediations. Third, we relied upon a nearly restricted sample of manufacturing employees, within which women are largely under-represented. Albeit this is consistent with the overall situation in Italy regarding gender distribution in industrial manufacturing organizations (ISTAT, 2013), future research might examine more equally distributed samples in order to consider possible gender differences. Fourth, the range of variables was limited. Future research should include, at least, both directions of work-family conflict and work-family enrichment. Finally, the outcomes’ amount of explained variance was low to moderate; future research should expand the range of potential FWE antecedents.

## Study 2

### Method

This study, as typical of the anthropological approach, focuses on qualitative, in-depth analysis, constructed on storytelling and long interviews.

Twenty in-depth interviews (10 male and 10 women, age 37-47, with junior- or high-school degree, married with 1 or 2 children) were carried out among the factory blue-collar workers, via a snow-ball sampling. The anthropological semi-structured interviews used in this study featured a track with several open questions; participants were also free to suggest the inclusion of issues or aspects considered important. The interviews’ track featured three main thematic blocks: *work life, family life and children care, work-family conciliation strategies*. Examples of questions were: would you describe your work activities [work life]? Could you describe the organization of your work schedule (e.g., shiftwork, etc.) [work life]? Could you describe how, and if, the organization of your household activities interacts with your work schedule [family life]? Could you describe the sharing of household activities and children care between you and your partner [family life]? Could you describe which programs and instruments (kind of help/support) you recur to for conciliating between work and family [work-family conciliation strategies]? Could you describe if, and how, any of your family experience or competence is able to enrich your work life [work-family conciliation strategies]? The complete list of questions is available from the second author upon a request.

Anthropological interviews always involve ethnography, namely a participative practice based on several meetings, visits and observations of family settings, events and routines (Agar, 2010; Herzfeld, 2014). The possibility to meet the workers and their families outside the interviews’ set (e.g., for having a coffee, tea or dinner), was an opportunity for obtaining an observational perspective, facilitating the completion and enrichment of the interviews with some discrepancies or gaps between behaviors and stories.

The analysis of tales and texts was inspired by the interpretive methodology, focused on the cognitive system and the horizon of values that the subjects expressed answering the questions and describing their daily life and family behaviors (Geertz, 1973, 1983). In this way, the reconstruction of practices, obtained during the interviews, is able to enlighten real sides of the family life, particular strategies of conciliation, situations during which work and family intertwine and enrichment processes occur. The specificity of the anthropological approach consists therefore in the possibility to describe, in a qualitative way, parts and scraps of current situations and cultural horizon framing the choices of the family life.

### Results and Discussion

As introduced earlier, three different thematic blocks were identified in order to analyse the interview reports, namely: *work life, family life and children care*, and *work-family conciliation strategies*. Indeed, although clearly heuristically defined, these aforementioned topic areas do not seem substantially quite so separated and mutually exclusive, as some overlap does exist in the way individuals usually describe and represent their work- and family-experiences.

Regarding work characteristics and engagement, the first noticeable aspect emerging from the interviews concerned the shift work issue: despite the repetitive nature of the production line, individuals agreed that their work did not require much effort, but rather it was characterized by rigid working hours and the mechanical repetition of shifts (morning, afternoon, night); in fact, shift work entailed unavoidable absences from home, therefore resulting in the loss of essential moments of ordinary family life and child care (e.g., having lunch or dinner all together). Chores at home usually weighted on women, who complained about the distress in simultaneously juggling between household management, picking children from school, and cooking, with the extra difficulty that often they were not at home for lunch and/or dinner time.

In the framework of the Italian situation as described above, it is not difficult to understand how work-family conciliation can be somewhat problematic and difficult because of shift work, and yet it is up to women experiencing, together with the difficulties, a strong transformation in “traditional” family relationships that, as we said, are still geared toward a markedly unbalanced weight care. Nonetheless, it is fair to state that, despite the difficulties, significant levels of work motivation did persist. So, Sara, 32 years old, with a middle school educational level, married for a year and childless, tells:

My husband supports me a lot, in fact he says: ‘if you want to work, do it just for yourself’. With my savings I made my bath over, I painted my house, I bought a lot of stuff, I gave some of my own money to other people: I helped my mother after my father died, and I helped my sister too. (...) In the first place, my job allows me to live independently of my husband, meaning that my husband always contributes to our household expenses, but I if I wish to purchase a TV, I buy it with my own savings.

In the Italian context, where female employment rates are lower than in the rest of Europe, as well as the model of the male breadwinner is still dominant, having a job becomes fundamental for women’s emancipation, and in some cases also a way to delay the marriage or, at least, to not consider getting married as the best chance for a woman (condition that is still widespread in Southern Italy). It is the case of Maria, 45 years old, with a middle school educational level, married and with two children (6 and 4 years old):

I replaced my father. My father retired and I took his place. Since I was the eldest daughter, as well as not engaged and unemployed, my father asked me if I wanted to prove myself. ‘If you will be fine, great!’ – he said to me – ‘And even if it won’t work out, whatever. Besides, since you will begin working, you will be able to make some money. So, you will be fine, although if you won’t like the work so much. Because you know, one can’t live without money’.

Maria met her husband at the factory (he was one of her co-workers). They got married when she was 39, and now they have two children. Nonetheless, she still kept working, being fully aware that two incomes allowed for a higher purchasing power, as well as a better social status and lifestyle for her family.

Therefore, the economic self-sufficiency by means of work, coupled with the decision-making ability to manage money, was a concrete and meaningful milestone for women, which pushed and motivated them also in overcoming the difficulties of shift work and, at the same time, provided them with a greater ability to demand their partners a more active participation in the domestic sphere and a better balance in sharing housework. In fact, when both partners did work, this also incited husbands towards a growing commitment in the domestic sphere: Giulia, 37 years, two sons and a working husband, proudly told: ‘My husband does what her wife actually does’. In essence, the first aspect of enrichment and work-family conciliation that work entails is the greater availability of male workers in participating in housekeeping and, conversely, the increased readiness of women in requiring their partners a more equal domestic partnership.

Simultaneously, indeed, it is obvious from the interviews with male workers that they did express quite a satisfaction in participating housework, flaunting their supportive and collaborative role, and representing themselves as more “modern” as directly involved in work-family conciliation issues, traditionally considered an exclusively female problem.

As the Mediterranean model is characterized by a gendered division of domestic labour still heavily weighted on the side of women, factory work, despite all the difficulties related to the shift work, therefore appeared to women as a significant factor of emancipation and, more generally within couples, as a factor that encouraged a cultural change in order to offset the burden of domestic work. This had a positive effect on the satisfaction of women for their work role, and even men in dual-earner families were satisfied and engaged with their job, given that both in the couple felt producers of income as well as active protagonists of their family life.

In addition, women were also spokespersons for the gratification that the job offered through the experience of a broader horizon of life than the domestic one did, giving them the chance to meet people, gain experience, deal with various people and situations, with an inevitable positive impact throughout own existential path:

*I enjoy that at work you can know other people. There are more than 1200, 1300 workers* [authors’ note: a more correct estimate is 1000]*: you can always experience and learn something from them. You can also meet nice people, and perhaps they may help you on your life journey, enriching you with their experience. It means that you can understand something more in depth, or discuss it with them. This means a lot for me even on my private side, in respect to my family. One grows, too.* (Michela, age 32)

Workers therefore appreciated all the stimuli and possibilities for knowledge arising from the world outside the household that the work itself provided them, with a clear enrichment of the experiences and their meaning on the side of private and family life. In this way, work became a cognitive horizon filled with values, able to go beyond the boundaries of the work sphere strictly speaking and therefore becoming a key factor that gave a boost to expand the workers’ life horizon with a dynamic and flexible interchange between areas of life. This explains why, although work-family conciliation is difficult under the system of shift work, work became acceptable and necessary and, on balance, women did not think of retiring and returning to an exclusively domestic life situation, which would represent not only an economic involution, but also a more general retrograde step in their lifestyle’s improvement.

Our anthropological in-depth perspective allowed us to find qualitative evidence about the possibility that work, and especially female work, can have a positive effect on gender equality and family care organization; as a result, work motivation also increases for both male and female workers, and it can help to overcome some difficulties in daily family life. In a gendered perspective, it is worth mentioning that the women in our sample, as emerged from interviews, were able to work expressing more satisfaction and pleasure “with a little help” from their husbands, but also men became more able to collaborate and accomplish care tasks with enthusiasm and participation “with a little sustain” from their partners. It is an interesting interchange between work and family, based on material and psychological resources, able to strengthen each counterpart and thus producing a positive cultural change. This means, in the Italian situation, the possibility to realize a more equal distribution of household work between men and women, and a more advanced familiar model, in line with gender equality and a correct sharing of roles, inside and outside the family.

## Conclusions

The challenges faced by actual human resource professionals entail the difficulty of grasping the changing nature of the workforce, including a rise in dual-earner couples with burdensome childcare and eldercare responsibilities. As work and family domains are so much intertwined, maintaining a harmonious relationship between the two will benefit unavoidably both individuals and organizations (Orkibi & Brandt, 2015). Notwithstanding the limitations already mentioned, the two different studies constituting our research provided together a step further in understanding how these important life domains may influence each other. Keeping in mind for both studies the call of the extant literature that a deeper understanding of the work-family experience will not be fully achieved until researchers will not devote as much attention to enrichment as they devoted to conflict (Greenhaus & Parasuraman, 1999), in Study 1 we posited that different facets of family social support would be positively associated with FWE and family workload negatively; moreover we hypothesized that FWE would be positively associated, as a mediator, with work engagement and life satisfaction. We found that employees feeling emotionally supported by their family members experienced higher FWE (Wayne et al., 2006) which, moreover, acted as a mediator with regard to work engagement and life satisfaction (Mauno et al., 2011). Finally, we found that experiencing extra-household support (e.g., from relatives) positively impacted on life satisfaction (Fu & Shaffer, 2001). Such results on one side seem to disconfirm the negative role of family workload with respect of FWE (as we already said, a potential explanation may reside in our male-dominated sample), while on the other highlight the importance of family emotional support (as well as from extra-household sources). Thus, our findings corroborate the importance of support in line with previous research (e.g., Greenhaus & Powell, 2003; Radcliffe & Cassell, 2014) and challenges practitioners and HR professionals to pay attention to these variables in designing interventions that, through the improvement of the employees’ work-family interface, could also have positive effects for the organization. Besides, after decades of debate about whether the availability of support reduces work-family conflict, we go further reframing from the outset the relationship between work and life in terms of mutual enrichment. Plus, consistently with recent literature calls to recast the work-family duality introducing a ‘third space’ where workers can manage these domains through an active collaboration among multiple groups (organizations, families, churches, schools, recreation centers, and so on) (Putnam, Myers, & Gailliard, 2014), we begin to fill this scenario including characters outside the couple, such as elder relatives or neighbors, whose support can be helpful.

As for our in-depth qualitative Study 2, results depicted a more complex picture than the survey could not return. Male participation in family chores seemed mainly depending on women emancipation and their absences from home because of work. Moreover, female FWE dynamics seemed based on crossover processes, that is female work experiences pushed male partners, first under the pressure of daily needs, and then on the basis of a cultural change, to be more participative in family chores. From our interviews, on one side we observed FWE dynamics and their positive effects on both work and general life, but on the other, these dynamics seemed depending on a complex interplay that took into account processes of crossover, work-to-family enrichment and conflict. Indeed, reconnecting to a research framework that has overemphasized the complex and problematic aspects of work-family conciliation in the Italian case (Donati & Prandini, 2008; Dovigo, 2007; Garofalo & Marra, 2008), our Study 2 stressed both the unavoidable several difficulties faced by dual-earner couples, particularly by women who bear the main responsibility for housework, and the positive consequences of managing simultaneously multiple roles (e.g., wife, mother, worker). Although being a preliminary study, it leaves the door open to future research in continuing to examine the positive aspects of interplay and mutual enrichment that family and work arenas may have, in the framework of a stronger motivation to achieve positive work-family conciliation outcomes.

In conclusion, the combination of different methodologies would certainly be an interesting future pursuit in work-family literature; just as we have learnt a great deal about positive relationships between work and family through our quantitative first study, we could deepen our understanding of these same relationships through Study 2 qualitative analyses, which allowed for gaining insight into individual experiences, specifically how people perceived and interpreted their interactions within family and workplace, therefore showing an otherwise hidden interrelatedness between emotions and attitudes experienced in the various domains of life. In this way, we want to embrace a more open, flexible and complex concept of enrichment, comprising different and simultaneous dynamics, that seems better explicable with an integrative quanti-qualitative approach, able to describe and explain *real* processes of enrichment.

## Closing Comments

We have extensively outlined, both theoretically and empirically, the benefits stemming from combing multiple roles. However, part of the future impetus should be also directed to pragmatically develop a wide range of family-friendly programs, practices and social policies that support devotion to work and family spheres (Butts, Casper, & Yang, 2013). Currently, the extent and nature of these initiatives may vary widely across countries, yet generally they relate to care-related leaves, child-referral services, and flexible work arrangements (Hegewisch & Gornick, 2011). However, the mere availability of these discretionary provisions does not automatically guarantee their proper and extensive use. The cold truth is that too often “the policy exists but you can’t really use it” (Kirby & Krone, 2002), at least if workers do not want to be perceived as being less committed to the organization (Allen & Russell, 1999) or labelled as troublemakers who create additional work for others or receive unfair benefits at others’ expenses (Ryan & Kossek, 2008). If not legitimized by a work-family culture who wholeheartedly supports work-family balance, policies take-up may be indeed thwarted by employees’ fear of backlash and negative career consequences, such as lower rewards and fewer advancement opportunities (Butts et al., 2013; Thompson, Beauvais, & Lyness, 1999). In the long run, this would result in a pessimistic scenario very much alike that one sketched by Khallash and Kruse (2012), where work-life balance will end to be just a privilege for a small segment of employees whose policies’ use comes with minimal career costs, while would not exist such a thing for the majority of workers who struggle to hold on to their jobs. That lack of such a concern on the part of the organization for the worker’s well-being will unavoidably hamper not only the employee, increasingly in jeopardy in coordinating and managing multiple life roles, but mostly the organization itself, which will likely experience poor productivity, higher turnover occurrences and absenteeism (Allen, 2001). Employees’ families are key business stakeholders and their interest must be taken into account in management practices not only on the basis of ethical or common sense concerns but, given their influence on workers’ satisfaction and consequent motivation, because they can help making the difference in terms of organizational performance and efficiency. Therefore, implicit in this argument becomes that taking care of accommodating and integrating employees’ family needs is a crucial means for today enterprises for maintaining a competitive advantage in the turbulent economic environment they live, a strategic choice for attracting and retaining employees who otherwise will experience more and more tension between work and family lives.
